# Saturation effect and transmembrane conversion of *Monascus* pigment in nonionic surfactant aqueous solution

**DOI:** 10.1186/s13568-017-0327-z

**Published:** 2017-01-23

**Authors:** Gong Chen, Qi Bei, Kan Shi, Xiaofei Tian, Zhenqiang Wu

**Affiliations:** 1 School of Bioscience and Bioengineering, South China University of Technology, Guangzhou, 510006 People’s Republic of China; 2 Dongguan Tianyi Biotechnology Co. Ltd.,, Dongguan, 523000 People’s Republic of China

**Keywords:** *Monascus* pigment, Secretion limitation, Transmembrane conversion, Cell density, Nonionic surfactant concentration

## Abstract

Extractive fermentation in a nonionic surfactant aqueous solution provides a promising and efficient method to produce *Monascus* pigments. The behaviour of pigment secretion during the extractive cultivation was investigated in the present work. The results revealed that the secretion of intracellular pigment was limited by its saturation concentration in the nonionic surfactant aqueous solution. The intracellular pigment was completely extracted to the outside of the cell at a low cell density and high concentration of Triton X-100 (TX) in fermentation broth; otherwise, a restriction for pigment extraction would occur. The decrement of the intracellular orange and yellow pigments was inconsistent with the increment of extracellular pigments with an increase in the TX concentration. It could be inferred that the intracellular orange pigment was converted to extracellular yellow pigment during the transmembrane secretion process, which might be attributed to the enzyme catalysis in the non-aqueous phase solution. This study helps explain the mechanism of variation of pigment characteristic and extraction capacity in extractive fermentation.

## Introduction

*Monascus* pigments, which are the functional secondary metabolites synthesized by *Monascus* fungi, are a mixed group of azaphilones that contain yellow, orange and red compounds and have been widely used as promising pigment additives in food and pharmaceutical industries (Lin et al. 2008; Patakova 2013). In submerged fermentation, *Monascus* pigments are mainly synthesized and accumulated in the mycelia (Chen and Johns 1993), in which two orange pigments (rubropunctatin and monascorubrin), two red pigments (rubropunctamine and monascorubramine) and two yellow pigments (monascin and ankaflavin) have been identified as the main intracellular pigments (Feng et al. 2012). However, accumulation of the intracellular pigments trapped in the mycelia became a significant challenge for the higher productivity of the total pigments due to the feedback inhibition and production degradation (Hu et al. 2012a; Chen et al. 2015).

Extractive fermentation with micelles of the nonionic surfactant in an aqueous solution is an efficient method for eliminating product inhibition and preventing product degradation, which promotes the productivity of *Monascus* pigments (Wang and Dai 2010; Kleinegris et al. 2011). Compared with other nonionic surfactants such as Tween 80, Span 20, Triton X-114, Brij 30, PEG 4000, and Pluronic, Triton X-100 (TX) showed an excellent biocompatibility to fungal growth and extractive efficiency for *Monascus* pigment in an extractive fermentation (Hu et al. 2012a, b). By using TX as the additive, an enrichment of the hydrophobic pigments occurred upon being “milked” in the artificial nonionic micelle aqueous solution to improve the pigment penetration across the cellular membrane (Hu et al. 2012b; Kang et al. 2013a). Further study indicated that the nonionic surfactant may modify the cell membrane lipid layer and then improve the rate of secretion of intracellular hydrophobic pigments across the cell membrane (Wang et al. 2013). However, the final extracellular pigment concentration is mainly attributed to the solubilization of pigments into the nonionic surfactant micelles and then increasing the concentration gradient between the intracellular and extracellular pigments (Kang et al. 2013b).

It has been shown that a conversion among the yellow, orange and red *Monascus* pigments might involve the *MpigE* gene (Liu et al. 2014; Shao et al. 2014), although the biosynthesis pathway of pigment remains unclear or controversial (Yang et al. 2015). It has been reported that pH plays an important role in regulating the pigment components (Shi et al. 2015, 2016). Moreover, the pigment characteristic is varied in high-cell-density (Chen et al. 2015) and extractive fermentation (Kang et al. 2013b). Additionally, the pH, TX concentration and timing of the surfactant application showed significant influences in modulating the pigment metabolism and characteristics during extractive fermentation (Kang et al. 2014; Xiong et al. 2015). However, the mechanism of pigment conversion in the nonionic surfactant aqueous solution is unclear and has not yet been reported.

In this study, the extractive cultivation of mature cell, fermentation broth and pigment powder was performed in a nonionic surfactant micelle aqueous solution. The extractive characteristic of TX for *Monascus* pigments was also investigated. The responses of pigment conversion to the trans-membrane secretion and the variation of pigment yield and components were studied accordingly.

## Materials and methods

### Microorganism and culture

*Monascus anka* GIM 3.592 which is deposited in the publicly accessible culture collection GDMCC/GIMCC (Guangdong Culture Collection Centre of Microbiology, China) was maintained on potato dextrose agar medium (potato dextrose 200 g, glucose 20 g, and agar 15–20 g/L of distilled water) and preserved at 4 °C.

The inoculum culture medium consisted of glucose 20 g, yeast extract 3 g, peptone 10 g, KH_2_PO_4_ 4 g, KCl 0.5 g, and FeSO_4_·7H_2_O 0.01 g per litre of distilled water. The fermentation culture medium consisted of glucose 50 g, (NH_4_)_2_SO_4_ 5 g, KH_2_PO_4_ 5 g, MgSO_4_·7H_2_O 0.5 g, KCl 0.5 g, MnSO_4_·H_2_O 0.03 g, ZnSO_4_·7H_2_O 0.01 g and FeSO_4_·7H_2_O 0.01 g per litre of distilled water. The initial pH in the seed and fermentation media was natural. The inoculum culture and conventional batch culture were conducted using the same methods reported in our previous work (Chen et al. 2015).

### Fermentation broth extracted by different concentrations of nonionic surfactant

A certain amount of fermentation broth that had cultivated for 6 days was withdrawn and diluted 100 times with distilled water. Then, 5 mL of both the original fermentation broth and diluted fermentation broth was added to 0, 5, 40, and 160 g/L TX for 1 h of extractive cultivation at 30 °C with 180 rpm. Subsequently, the extractive broth was centrifuged at 10,000 rpm for 5 min to separate the mycelia, and the supernatants were diluted to determine the extracellular pigments concentration and composition. The mycelia were soaked in the same volume of 70% (v/v) ethanol aqueous solution (pH = 2) for 1 h to extract and determine the residual intracellular pigment concentrations and compositions.

### Mature cells extracted by different concentrations of nonionic surfactant

The fermentation broth that had been cultivated for 6 days was withdrawn and centrifuged at 10,000 rpm for 5 min. The collected wet mycelia were washed three times with distilled water and divided into three groups of 0.01, 0.1, and 0.25 g. Then, they were soaked in 5 mL of 0, 5, 40, and 160 g/L TX aqueous solutions, respectively, and then incubated at 30 °C with 180 rpm for 1 h. Subsequently, the extractive broths were centrifuged at 10,000 rpm for 5 min to separate the mycelia, and the supernatants were diluted to determine the extracellular pigments concentration and composition. The mycelia were soaked in the same volume of 70% (v/v) ethanol aqueous solution (pH = 2) for 1 h to extract and determine the residual intracellular pigment concentrations and compositions.

### Repeat extraction of mature cells in nonionic surfactant aqueous solution

Five millilitres of the fermentation broth that had cultivated for 6 days was added to 40 g/L TX for 1 h of extractive cultivation at 30 °C with 180 rpm. The extractive broth was centrifuged at 10,000 rpm for 5 min to separate the mycelia, and the supernatants were diluted to determine the extracellular pigments concentration for the first time. The mycelia were then soaked in the 40 g/L TX aqueous solutions to keep the same volume. After 1 h of incubation at 30 °C and 180 rpm, the supernatants were again separated to determine the extracellular pigments concentration for a second time. Next, the above operations were repeated to continuously determine the extracellular pigments concentration for a third and fourth time. Subsequently, the mycelia were collected and then soaked in the same volume of 70% (v/v) ethanol aqueous solution (pH = 2) for 1 h to extract and determine the residual intracellular pigment concentrations. Additionally, the extracellular and intracellular pigments in the original fermentation broth without extraction were separated and diluted to determine their concentrations. All extracted extracellular and intracellular pigments were also detected by TLC analysis to observe the difference.

### Pigment powder dissolved in nonionic surfactant and ethanol aqueous solution

The production process of pigment powder was as follows: the mycelia (100 g) that had been cultivated for 6 days were collected and washed three times with distilled water, then soaked in 1.5 L ethanol solution (pH = 2) for 4 h for extraction. The mycelia were filtered and repeat extracted, and the twice extraction broth was collected and concentrated using a rotary evaporator XHRE-52C (Xiaohan, Shanghai, China) at a low temperature. The concentrated colloidal solid pigment was taken out and dried in a low-temperature vacuum DZF-1 (Yetuo, Shanghai, China) to obtain the pigment powder.

The 0.01, 0.1, 0.4, and 0.8 g pigment powders were soaked in 25 mL of 40 g/L TX aqueous solutions, respectively, and cultivated at 30 °C and 180 rpm for 1 h. The supernatant was collected for measuring the pigment concentration by centrifuged at 10,000 rpm for 5 min. Moreover, the same content of 0.1 g pigment powder was extracted by 25 mL 5, 40, 160 g/L TX aqueous solution and 70% (v/v) ethanol solution (pH = 2), respectively. The supernatant was also collected to detect the pigment concentration.

### Pigment concentration and composition analysis

*Monascus* pigment concentrations are usually evaluated by their integrated color characteristics. The estimation of pigment concentrations in this study followed the same method that was detailed in our previous work (Chen et al. 2015; Shi et al. 2015, 2016). The concentrations were estimated from the visible spectrum and represented as absorbance unit (AU, multiplication of the absorbance with its dilution ratio for a certain sample). The TLC analysis described in previous work (Chen et al. 2015; Shi et al. 2015) was performed on the Silica gel 60 F254 TLC plate (Merck) and run with methylbenzene/ethyl acetate/formic acid (7:3:1) as the solvent system.

The pigment composition in different cultivation modes was also detected by a high performance liquid chromatography (HPLC) method. The chromatographic system consisted of a Waters e2695 Solvent Delivery Pump (Waters, USA) and a PDA-2998 UV-detector (Waters, USA). The mobile phase consisted of eluent A (water: phosphoric acid = 10,000:3, v/v) and eluent B (acetonitrile) at a flow rate of 1.000 mL/min, and the elution gradient was as follows: 0 min, 80% A and 20% B; 25 min, 20% A and 80% B; 35 min, 20% A and 80% B; 36 min, 80% A and 20% B; and 40 min, 80% A and 20% B. The detection temperature was at 30 °C and the detection wavelength was set to 410 nm.

### Statistical analysis

The data are expressed as the mean values ± standard deviation (SD) for each measurement. The data were submitted to an ANOVA analysis, and the significance of differences was determined by Duncan’s multiple range tests where necessary. *p* < 0.05 was considered statistically significant in all cases. All the analyses were carried out with the SPSS software package (version 22.0, SPSS Inc., Chicago, IL, USA).

## Results

### Saturation of pigment in fermentation broth with nonionic surfactant

**Fig. 1:**
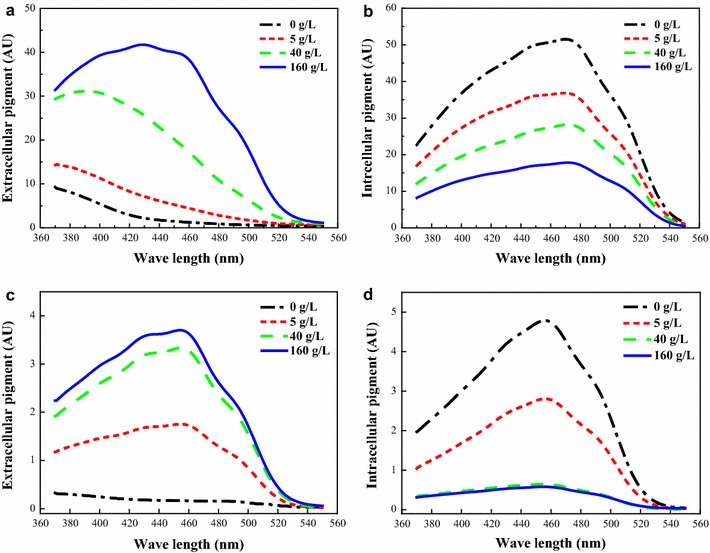
Pigment extracted from the fermentation broths by adding different concentrations of Triton X-100. **a**, **b** represent the extracellular and intracellular pigments extracted from the original fermentation broth, respectively. **c**, **d** represent the extracellular and intracellular pigments extracted from 100-fold dilution of the original fermentation broth, respectively

By adding TX, the amount of intracellular pigments in the mycelia decreased for extraction of the pigments to the extracellular broth (Fig. 1b), and the spectrum of intracellular pigment remained unchanged with a peak absorbance of orange pigment at approximately 470 nm (Zheng et al. 2009). Meanwhile, the extracellular pigment was significantly increased, and the spectrum was obviously varied. There was a unilateral decline curve from 370 to 550 nm at a low TX concentration of 5 g/L, similar to the conventional batch culture (Shi et al. 2015), but the curve changed and had the peak value at 390 nm at a high TX concentration of 40 g/L, which is the characteristic spectrum of yellow pigment (Zheng et al. 2009). When the TX concentration was increased to 160 g/L, the peak wavelength of the extracellular pigment was shifted to approximately 430 nm (Fig. 1a). Additionally, another small absorption peak could be found at about 460 nm, which might be attributed by orange pigments (Zheng et al. 2009). This result indicated that the extracellular pigment characteristic was shifting from yellow pigments to orange pigments during the trans-membrane secretion process under relatively low nonionic surfactant concentrations. To further investigate the characteristic variation of the pigment during extraction, the original fermentation broth was diluted 100-folds. The results showed that the intracellular pigment could nearly be extracted completely at a high TX concentration of 40 or 160 g/L (Fig. 1d) and that the spectrum of the residual intracellular pigment kept a stable peak wavelength at approximately 460 nm with an increasing concentration of TX (Fig. 1d). Yield of the extracellular pigment also remained at a high level in the high TX concentration (Fig. 1c). Based on the data above, it is concluded that there is an extractive limitation by TX saturation in the fermentation broth, which is related to the mycelia density.

### Saturation of pigments extracted from mature cells in nonionic surfactant aqueous solution

**Fig. 2:**
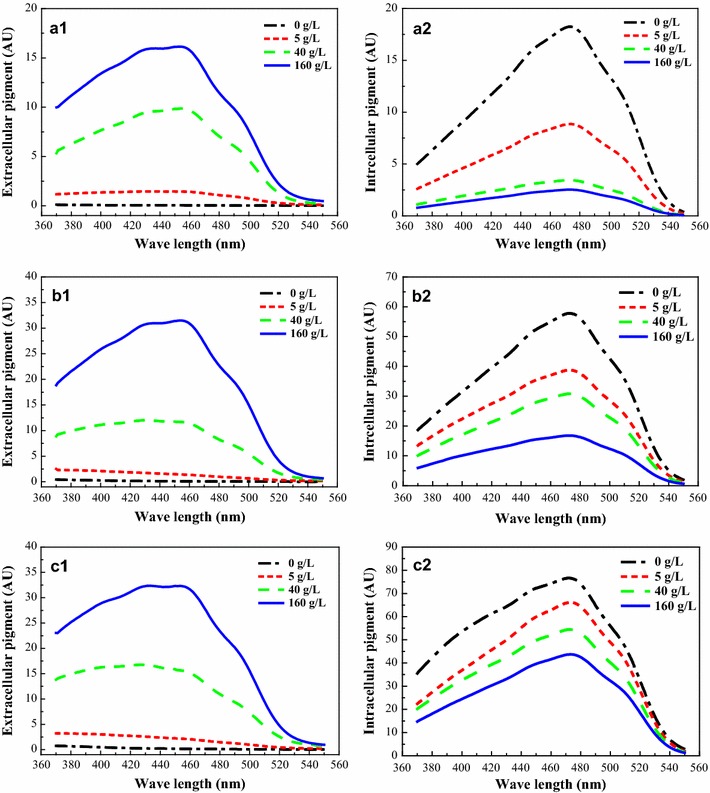
Pigment extracted from the mature cells by adding different concentrations of Triton X-100. **a1**, **b1**, **c1** represent the extracellular pigments extracted from 2, 20, 50 g/L mature cells, respectively. **a2**, **b2**, **c2** represent the intracellular pigments extracted from 2, 20, 50 g/L mature cells, respectively

By putting a low density of mature cells (2 g/L) in a TX aqueous solution, the intracellular pigments were extracted and gradually decreased as the TX concentration increased (Fig. 2, a2). Nearly 90% of the intracellular pigments could be extracted to the extracellular space when the TX concentration reached 160 g/L (Fig. 2, a2). However, the residual intracellular pigments were notably increased when with a high density of mature cells (20 g/L), even at a high TX concentration of 160 g/L (Fig. 2, b2). If the density of mature cells was further increased to 50 g/L, less than half of the intracellular pigments could be extracted to the extracellular space (Fig. 2, c2). This result indicated that the secretion of intracellular pigments was limited by the saturation of TX. It is worth noticing that the increment of extracellular pigments was inconsistent with the decrement of intracellular pigment at low TX concentration (5 g/L), but the difference did not exist at higher TX concentration (>40 g/L).

Using different concentrations of TX aqueous solutions, the ultraviolet (UV) spectra of the intracellular pigments at three densities (2, 20, and 50 g/L) of mature cells were almost the same (Fig. 2, a2–c2). All of the intracellular pigment solutions showed a single peak at wavelength of approximately 470 nm, which is the characteristic spectrum of orange pigment (Zheng et al. 2009). In contrast, the spectrum of the extracellular pigment regularly changed with different TX concentration (Fig. 2, a1–c1). With a low density of mature cells (2 g/L; Fig. 2, a1), the peak absorbance of extracted extracellular pigment switched to approximately 460 nm in the broth containing 5 g/L TX. The spectrum was quite distinct from the pattern of an unilateral decline curve between 370 and 550 nm in the conventional batch culture (Shi et al. 2015). It was worthy to note that the wavelength of peak absorbance gradually shifted from 390 to 460 nm when increasing TX concentration was applied from 5 to 160 g/L with a higher density of mature cells (20 g/L; Fig. 2, b1). This variation tendency of the peak wavelength induced by increasing TX concentrations became more obvious when the density of mature cells was increased to 50 g/L (Fig. 2, c1). These results indicated that the pigment characteristic could be alternated during the extraction process with different concentrations of the nonionic surfactant.

### Pigment differences following repeated extraction from mature cells with nonionic surfactant aqueous solution

**Fig. 3:**
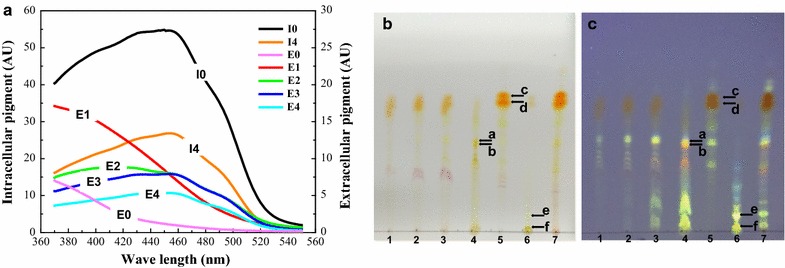
Pigment extraction and TLC analysis with repeat extraction of mature cells in Triton X-100 aqueous solutions. **a** Characteristic variations of intracellular and extracellular pigments. **b**, **c** TLC atlas in visible and ultraviolet light. *Lane 1*–*4*: extracellular samples with four repeated extractions (from 4 to 1); *Lane 5*: intracellular samples after four repeated extractions; *Lane 6*: extracellular samples without extraction; *Lane 7*: intracellular samples without extraction;* a*, *b* represent the intracellular *yellow pigments*;* c*,* d* represent the intracellular *orange pigments*;* e*,* f* represent the extracellular *yellow pigments*

The intracellular pigment was approximately 55 AU_460_/mL before extraction, and it remained at a high residual level after four batches of repeated extractive cultivation (Fig. 3A, I0 and I4). This indicated that a limitation existed for extracting the intracellular pigment to the outside by the saturated concentration of TX. The spectrum of the extracellular pigment showed that the yellow pigment was transported more during the first batch of the extraction because the peak wavelength of the spectrum was approximately 380 nm (Fig. 3A, e1). Then, the peak wavelength of the extracellular pigment gradually switched from 380 to 410 nm and to 460 nm for the second, third, and four batches of the extractive cultivation, respectively, which indicated that the orange pigment gradually became the dominated pigment as the number of repetitions increased (Fig. 3A, e2–e4). This result indicated that the pigment’s characteristics were altered in the repeated extraction process. Furthermore, it could be speculated that a conversion might exist between intracellular and extracellular pigments, according to the discrepancy between the increment of the extracellular pigments and the decrement of intracellular pigments.

Thin layer chromatography (TLC) showed that the intracellular pigment bands before and after extraction were mainly dominated by two common orange pigments (Fig. 3C, c and d). However, the brightness in the latter was weak, which illustrated that some of the orange pigments were transported to the outside after extractive cultivation (Fig. 3B, lanes 5 and 7). Nearly no orange pigments bands could be seen, but a relatively high brightness of the yellow pigment bands was present in the first batch extract, which implied that the yellow pigments were the dominant pigment in the extracellular broth (Fig. 3B, lane 4). In the following two repeated batches of extractive cultivation, the brightness of the yellow pigment bands gradually decreased, whereas the orange pigments relatively increased in contrast (Fig. 3B, C, lanes 2 and 3). After the fourth batch of extractive culture, almost no yellow pigments and only certain orange pigments were extracted (Fig. 3B, C, lane 1). Therefore, this further proved that there was an extractive saturation of pigment by TX, and the pigment characteristic was changed in the repeated extractive cultivation. Moreover, the intracellular yellow and orange pigments might be converted during the extraction process of trans-membrane secretion.

### Solubility and stabilization of pigments in a nonionic surfactant aqueous solution

**Fig. 4:**
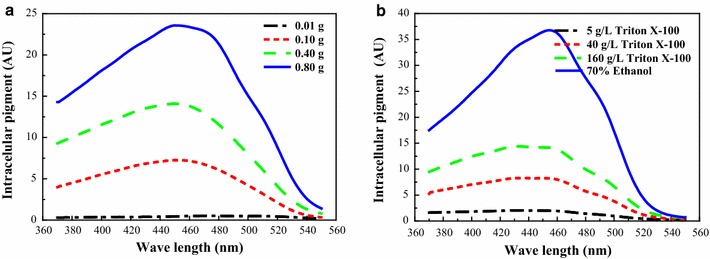
Intracellular pigment powder dissolved in different concentrations of nonionic surfactant. **a** 40 g/L Triton X-100 with different weights of pigment powder. **b** 0.1 g pigment powder in different concentrations of Triton X-100

When the pigment powder was soaked in the 40 g/L TX aqueous solutions, the yield of the extracellular pigment significantly varied with the amount of additive (Fig. 4a). The higher pigment powder content was, the more pigment could be extracted. The absorption peak remained unchanged at approximately 470 nm, which is the characteristic peak wavelength of orange pigments (Zheng et al. 2009). This indicated that the relative proportion of yellow and orange pigment compositions extracted by TX was the same, even with different content of pigment powder. Additionally, when 0.1 g of pigment powder was soaked in different concentrations of the TX aqueous solution, the pigment characteristic was unchanged, with a peak wavelength at approximately 470 nm (Fig. 4b), which implied that the extracted pigments were dominated by orange pigments. The peak of the absorption spectrum for the pigment powder extracted by 70% (v/v) ethanol aqueous solution (pH = 2.0) was also located at 470 nm, similar to that of the intracellular pigment in the raw mycelia, whereas the peak value corresponding to pigment concentration was much higher, thus showing a good extraction ability compared to the nonionic surfactant TX (Fig. 4b). These results indicated that there is an extractive saturation of TX micelle aqueous solution that is also correlated to the pigment concentration in the mycelia. Interestingly, the absorption spectrum of the extracted pigment was not altered, even with higher content of the pigment powder or when higher concentrations of nonionic surfactant were applied, compared to the fermentation broth or mature cells extracted. This result demonstrated that the pigment characteristic of the pigment powder was not shifted during the process of extraction by the TX aqueous solution.

### Transmembrane conversion of pigments in a nonionic surfactant aqueous solution

**Fig. 5:**
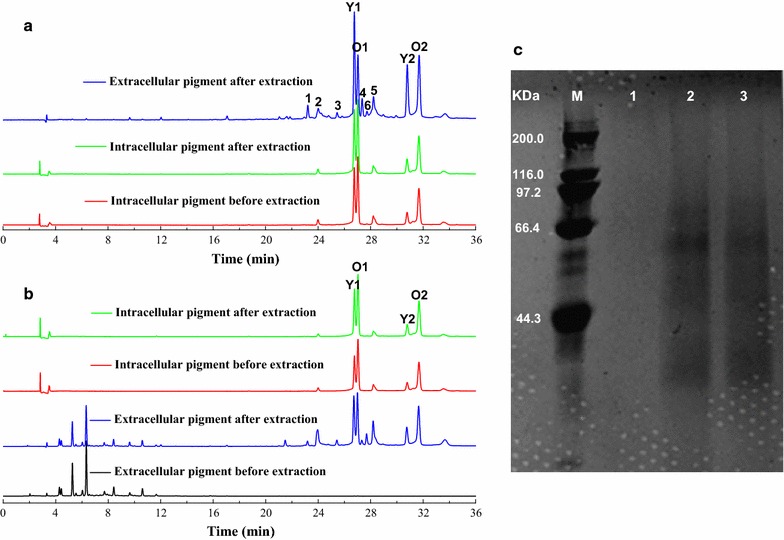
Pigment variation and protein secretion in nonionic surfactant aqueous solutions from mature cells and fermentation broth. **a** intracellular and extracellular pigments extracted from mature cells. **b** intracellular and extracellular pigments extracted from fermentation broth. **c** protein content and composition in extracellular broth with different extractive cultivations. M: marker; *1* conventional batch culture (not extracted), *2* fermentation broth extracted, *3* mature cells extracted

Variations of the intracellular and extracellular pigments in the nonionic surfactant micelle aqueous solutions were further investigated using HPLC. The results showed regardless of which fermentation broth or mature cells were used in the extraction process with different concentrations of TX, the residual intracellular pigment characteristics inside the mycelia were not changed (Fig. 5a, b, peak 2, Y1, O1, 5, Y2, O2). However, the extracellular pigment consisted of both the original (Fig. 5a, b, peak 2, Y1, O1, 5, Y2, O2) and newly generated (Fig. 5a, b, peak 1, 3, 4, 6) compositions of the extracted intracellular pigment. The new generated pigments possessed characteristic spectra of yellow pigments with the peak absorbance at 415, 429, 410 and 431 nm, respectively. Moreover, this variation appeared simultaneously in different concentrations of TX used, which indicated that the intracellular pigment might be converted in the process of being extracted to the outside. Additionally, the four main intracellular pigments, e.g., two yellow pigments (Y1-Monascine, Y2-Ankaflavine) and two orange pigments (O1-Rubropunctatine, O2-Monascorubrine), occupied a large proportion of the total pigments (Fig. 5a, b). Moreover, they were significantly (*p* < 0.05) changed during the trans-membrane secretion progress in the extractive cultivation of both mature cells and the fermentation broth. Interestingly, the ratio of the relative content of the two orange pigments to the two yellow pigments intracellularly (O/Y, In) remained unchanged with an increased TX concentration added into the both mature cells and fermentation broth, whereas the ratio of the relative content of the two orange pigments to the two yellow pigments extracellularly (O/Y, Ex) was significantly (*p* < 0.05) improved (Table 1). In contrast, the ratio of O/Y (Ex) was significantly (*p* < 0.05) improved from 1.93 to 33.37 with the increase of pigment powder extracted, which corresponded to when a decreased concentration of TX was applied. In addition, there was no newly generated pigment in the extracellular broth from all extractions with different TX concentrations. These results indicated that the intracellular yellow and orange pigments were secreted to the outside broth at the same proportion but that the dominant extracellular pigment extracted from the mycelia presented selective changed from yellow to orange pigment with an increase in the TX concentration. This change might be due to the conversion of orange pigments to yellow pigments during the trans-membrane secretion.

**Table 1:** Pigment variation in the extractive cultivation with different concentrations of nonionic surfactant aqueous solutions

Triton X-100 concentration (g/L)	Fermentation broth extracted		Mature cells extracted	
	O/Y(Ex)^a^	O/Y(In)^b^	O/Y(Ex)^a^	O/Y(In)^b^
0	–	2.09 ± 0.06	–	1.71 ± 0.09
5	0.82 ± 0.01a	2.03 ± 0.19	1.01 ± 0.07a	1.71 ± 0.15
40	1.58 ± 0.03b	2.05 ± 0.03	1.47 ± 0.01b	1.73 ± 0.07
160	1.78 ± 0.02c	1.97 ± 0.10	2.72 ± 0.05c	1.74 ± 0.14

The data are expressed as the mean values ± standard deviation (n = 3). Mean values in a column with different lowercase letters are significantly different (ANOVA, Tukey’s test; *p* < 0.05)

^a^Rate of extracellular two orange pigments to two yellow pigments

^b^Rate of intracellular two orange pigments to two yellow pigments

The content and composition of proteins in the extracellular broth extracted from the fermentation broth and mature cells were detected by sodium dodecyl sulphate polyacrylamide gel electrophoresis (SDS-PAGE) (Qu and Zhu 2016). The results showed nearly no protein bands in the extracellular broth of the conventional batch culture (Fig. 5c, line 1), while certain amount of intracellular proteins with obvious bands were extracted when the nonionic surfactant TX was added to the fermentation broth or mature cells (Fig. 5c, lines 2 and 3). These protein bands extracted from the fermentation broth or within mature cells were basically the same. As the pigment components extracted from the fermentation broth and mature cells were also remained unchanged (Fig. 5a, b), it could be induced that the transformation of pigment components in the extractive cultivation might be catalyzed by the intracellular enzymes that were relating to pigment conversion.

## Discussion

The production of intracellular *Monascus* pigments by submerged culture has been studied extensively, and extractive cultivation was found to be a good strategy to facilitate the secretion of intracellular pigments to the extracellular broth through trans-membrane transport (Hu et al. 2012a; Kang et al. 2013a). It was found that the accumulation of extracellular *Monascus* pigments was positively correlated with surfactant concentration and that higher concentrations of surfactant could extract more extracellular *Monascus* pigments (Xiong et al. 2015; Wang et al. 2016). In this work, only part of the intracellular pigment in the mycelia could be extracted to the outside broth even with a high TX concentration (Figs. 1b, 2b2, c2). Although repeated extraction of mature cells led to more intracellular pigment secretion, a certain amount of pigments was still restricted in the mycelia (Fig. 3a). It has been reported that the intracellular lipid droplets may have acted as reservoirs of the hydrophobic *Monascus* pigments and be limited by its space. In extractive cultivation, the nonionic surfactant might play a similar role as lipid droplets in store the extracted pigments by forming the aqueous micelle in the extracellular broth (Wang et al. 2015). Thus, a limit in extractive saturation for the secretion of intracellular pigments to the outside was seen in the nonionic surfactant micelle aqueous solutions.

The extraction of hydrophobic intracellular pigments to the extracellular broth followed a principle of equation (Koley and Bard 2010), and the secretion efficiency was highly related to the differences of pigment concentration between the two sides of cell membrane as well as the properties variation of the membrane (Kang et al. 2013a, b). This is consistent with the fact that higher concentrations of mature cells or fermentation broth, corresponding to higher amount of intracellular pigments, could be extracted higher extracellular pigments (Figs. 1a, 2c1). The repeated extraction of mature cells could lead to more intracellular pigment secretion when a concentration difference exists between intracellular and extracellular pigments (Fig. 3a). Moreover, higher content of pigment powder led to higher pigment concentrations after soaking in 40 g/L TX aqueous solutions (Fig. 4a). It has been reported that the lipid cell membrane could be modified in the nonionic surfactant micelle solution, thereby increasing the permeability of cell membrane and the intracellular pigment export in the perstractive fermentation process (Wang et al. 2013). In this study, with low pigment content in fermentation broth or mature cells, the intracellular pigments could be completely extracted into the extracellular broth (Figs. 1d,  2a2). In contrast, it was hard to have all the intracellular pigments extracted when their content was in higher levels (Figs. 1b, 2b2, c2). It has been reported that the nonionic surfactant has two polarities, hydrophilic and hydrophobic (Wang 2011), and that the intracellular hydrophobic pigment could be transported through the cell membrane to the extracellular nonionic surfactant micelles (Hu et al. 2012a; Kang et al. 2013a). There might be a relationship between pigment solubility in the nonionic surfactant micelles, such as with certain molecules of TX embedded in one pigment molecule. Therefore, the secretion of intracellular pigments to the outside in extractive fermentation depended primarily on the concentration of the nonionic surfactant applied, which could influence the cell membrane permeability as well as the pigment transportation and solubility.

Pigment degradation in the intracellular limited space has been reported (Xiong et al. 2015). Similarly, it was observed that the instability of extracellular pigment could cause a low recovery pigment yield in the extractive fermentation broth with a low surfactant concentration. However, little degradation of extracellular pigment occurred when high concentrations of nonionic surfactant were applied (Kang et al. 2013a; Xiong et al. 2015). The similar results obtained in our study showed that the decrease in intracellular pigment was not equal to the increase of extracellular pigment in low concentrations of TX, but not in higher TX concentrations (Figs. 1,  2). In addition, there were many new components (mainly yellow pigments) in the extracellular broth after extractive cultivation compared to both the conventional intracellular and extracellular pigment compositions (Fig. 5a, b). It was also found that the spectrum characteristic of extracellular pigments varied at low or high concentrations of TX aqueous solutions (Figs. 1, 2, 3); even the intracellular yellow and orange pigments were extracted at the same proportion in fermentation broth or mature cells (Table 1). TLC analysis also showed the differences of pigment components from various stage of repeated extractive cultivation (Fig. 3b, c). As an extractive microbial transformation in nonionic surfactant micelle aqueous solution was reported (Ye et al. 2004; Xue et al. 2010), it was indicated that there was a conversion of the intracellular yellow and orange pigments during the extraction process. The orange pigments could be transformed into yellow pigments by chemical hydrogenation catalyzed by related enzymes (Carels and Shepherd 1977; Hajjaj et al. 2000). What’s more, the hydrophobic intracellular pigments were generally accumulated in vacuole or lipid droplets (Wang et al. 2015) that may isolate the pigments from catalysis by enzymes that gathered in other granular inclusions. Therefore, the related enzymes involved in the catalytic conversion of orange pigments to yellow ones might have been simultaneously extracted with the intracellular pigments into the TX aqueous solutions. This was supported by the fact that obvious protein bands were presented in the extracellular broth by SDS-PAGE after extractive cultivation (Fig. 5c). Furthermore, the transmembrane conversion was also supported by the unchanged characteristic of the pigment from the pigment powder extracted with different concentrations of TX because there were no enzymes and only the extraction process of the pigment powder (Fig. 4).

Additionally, the yellow pigments with a relatively low polarity were more easily secreted into the extracellular TX micelle aqueous solution due to the high solubility in the surfactant micelles (Wang et al. 2016). Moreover, the orange pigments showed less stability and could be transformed to red and yellow pigments (Chen et al. 2015; Shi et al. 2015, 2016; Chen and Wu 2016). Therefore, the relatively low TX concentration led to the degradation of orange pigments as well as the conversion from orange to yellow pigments, which resulted in the yellow pigment being dominant in the extracellular broth (Figs. 1a, 2b1, c1). However, the relatively high TX concentration could limit the degradation of orange pigments (Wang et al. 2015) and extract large amount of intracellular pigments (mainly orange pigments) to the extracellular broth, so the orange pigment still occupied a large proportion, even though a certain amount was converted from orange pigments to yellow pigments (Figs. 1c, 2a1). Furthermore, the repeated extraction of mature cells showed that there was a conversion in the first batch due to the related enzyme being secreted to the extracellular broth and catalyzing the non-aqueous phase reaction. The spectrum of extracellular pigments in the third and fourth extractions presented the characteristics of orange pigment, which indicated that no conversion existed because there was no residual enzyme within the mycelia that could be extracted to the outside (Fig. 3a). Therefore, the pigment characteristic had changed during the extraction process in the TX micelle aqueous solutions, which was mainly due to the conversion of pigments rather than to instability.

## Abbreviations

TLC: thin layer chromatography
HPLC: high performance liquid chromatography
TX: Triton X-100
SDS-PAGE: sodium dodecyl sulphate polyacrylamide gel electrophoresis
